# Personal

**Published:** 1855-05

**Authors:** 


### EDITORIAL DEPARTMENT.

*Personal.* By the SENIOR EDITOR.

It will be perceived by reference to the prospectus for the next volume of the BUFFALO MEDICAL JOURNAL, that the connection of the senior editor ceases with the present number. My reasons for retiring from farther editorial labors are, simply, that they interfere with duties and occupations which claim precedence, and I am unwilling, as has been the case of late, to continue merely a nominal editor. The Buffalo Journal, with the conclusion of this volume, completes the first decade of its existence. It was commenced ten years ago as a monthly of twenty-four pages. At the expiration of the first year it was increased to its present size, and has remained without change up to this time. The publisher, at the beginning, was Mr. C. F. S. Thomas, a gentleman distinguished for his knowledge and skill in matters pertaining to the typographical art, and widely known by his enterprise and success in that line of business. It is but justice to him to state he engaged in the publication with no immediate expectation of pecuniary profit, prompted solely by a disposition to coöperate in the undertaking, and, I may add, friendly consideration for the editor. It has remained in his hands, in connection with those with whom he has been associated in business, until now, and I am happy in saying that during ten years of intercourse as editor and publishers, the pleasure of the relation with all parties, so far as I know, has been undisturbed by the slightest unpleasant circumstance. The increase in the subscription list has steadily gone on year after year, and this almost without any efforts having been made to increase its patronage by advertising, or personal solicitations. For its present circulation it is indebted to no extrinsic influences, but exclusively to its own recommendations to the favorable regard of the medical profession. This statement, I trust, will be deemed excusable when I add, I am well aware that whatever merit it has been thought to possess, has been derived in a great measure from its collaborators. To these I avail myself of this opportunity to tender my grateful acknowledgments. The amount of matter, however, contributed by the editor has been large, and it would be affectation not to confess the satisfaction of believing that, as a whole, it has contributed to the success of the work. Certainly if I had not felt encouraged by presuming to entertain such a belief, I should long since have vacated the editorial chair.

Ten years' experience may not seem sufficient to authorise much pretension on the score of a long career of editorship, but in view of the changes that have taken place, in that length of time, among contemporaneous journals, I have some claim to be considered a veteran in the service. Of sixteen medical periodicals which I find on consulting the exchange list when this journal was commenced, eleven are still in existence; and of the editors at that time connected with them, I believe I am correct in saying but two besides myself have kept their places uninterruptedly on the tripod. The persons referred to are Dr. Isaac Hays and Prof. Lunsford P. Yandell. Meanwhile several journals have come into being, and some of the latter have lived and died within that period. I should do injustice alike to my own feelings, and my brethren of the *corps editoriale*, if I omitted to acknowledge the courtesy uniformly extended by them to the-Buffalo Medical Journal. I cannot recall a single instance in which any difference or controversy has occurred, involving other than kind and friendly feelings.

At the risk of incurring the charge of egotism, I shall not close this article without briefly adverting to the course which I have pursused as a medical editor. In expressing opinions relating to books, institutions, education, and other matters of interest to the profession, I have constantly aimed to be impartial and independent. As to the views that have been expressed on various subjects I have nothing now to say, save that I am very far from wishing to assume them to have always been correct; nor have I any disposition to claim exemption from prejudices to which all minds are more or less exposed; but this I will say, that, after as candid a retrospection as I am able to make, I do not discover ends and motives, the recollection of which occasions self-reproach. I have not intentionally done injustice to any one, nor am I conscious of having in any instance transcended the limits of fair criticism, and gratuitously wounded the feelings of others. It is difficult, if not impossible, in conducting a public journal, to steer altogether clear of difficulties, and meet the wishes of all. That I have in some instances given dissatisfaction, I know; perhaps this has been the case in more instances than have come to my knowledge. They, however, who may have taken um brage at my editorial remarks, with very few exceptions, have been considerate enough to leave me in ignorance of the fact, for which, if this article should chance to meet their eyes, they will please accept my thanks.

The permanency of the Buffalo Medical Journal, so far as it may be predicated on its present patrons and friends, is secure. It is in a position, not only to warrant its continuance, but to afford an income which will be in some measure remunerative to its proprietor. In this fact there is a guaranty of stability for the future, and also a protection against diminution of the zeal and vigor with which it will be conducted. Few persons are able to find it consistent with their obligations to themselves and others to labor gratuitously for an unlimited period, and, assuredly, there is no good reason why the services of a medical editor should be rendered without pecuniary recompense.

In relinquishing the Journal to my successor it is quite needless for me to say, that its prospective character and usefulness are not left to conjecture. The subscribers are aware that the editorial duties for the past year have entirely devolved upon my associate who now becomes sole editor and proprietor. The cont'nued increase in its circulation furnishes satisfactory proof, in addition to the evidence which every reader of the work can appreciate, of the industry and ability with which, during the time just mentioned, it has been conducted.

And now, resuming for the last time, in this last paragraph, the editorial plural, with renewed thanks to those who have contributed to its success, and with our best wishes for its prosperity in years to come, we take leave, as editor of the Buffalo Medical Journal. A. F.

